# Frequency and Risk Factors for Persistent and Concomitant Symptoms after Hospital Discharge for Coronavirus Disease 2019 in the Pre-omicron Period: An Exploratory Longitudinal Study

**DOI:** 10.31662/jmaj.2023-0054

**Published:** 2023-09-27

**Authors:** Taketsune Kobuchi, Hidenori Onishi, Osamu Yamamura, Ippei Sakamaki, Hiromichi Iwasaki, Hiroyuki Hayashi

**Affiliations:** 1 Department of Emergency, University of Fukui Hospital, Fukui, Japan; 2 Department of Community Medicine, School of Medical Sciences, University of Fukui, Fukui, Japan; 3 Department of Infectious Diseases, School of Medical Sciences, University of Fukui, Fukui, Japan; 4 Department of Infection Control and Prevention, University of Fukui Hospital, Fukui, Japan; 5 Department of Family Medicine, University of Fukui Hospital, Fukui, Japan

**Keywords:** Patient Discharge, Cough, Logistic Models, Japan, Coronavirus disease of 2019, Fatigue, Risk Factors, Muscle Weakness

## Abstract

**Introduction:**

Many countries have reported persistent and concomitant symptoms of coronavirus disease 2019 (COVID-19). This study aimed to identify persistent COVID-19 and concomitant symptoms in discharged patients and identify the risk factors for such symptoms.

**Methods:**

This study enrolled patients with COVID-19 who were admitted to the University of Fukui Hospital, Japan, and discharged between April 3, 2020, and August 19, 2021. Persistent and concomitant symptoms were confirmed based on medical examinations approximately 2 weeks after discharge. Patient characteristics and symptoms were collected from the patients’ medical records by a technical assistant.

**Results:**

This study included 120 patients (60 men and 60 women; mean age, 53.5 ± 17.0 years). Persistent COVID-19 symptoms were observed in 62 patients (51.7%). The most common persistent symptom was weakened physical function, manifesting as physical weakness (48.4%) and muscle weakness (29.0%). Binary logistic regression analysis revealed that cough with expectoration within the acute phase of COVID-19 was a risk factor predisposing patients to COVID-19 sequelae (odds ratio: 2.94, 95% confidence interval: 1.300 - 6.630, *p* = 0.009).

**Conclusions:**

The study findings suggest that productive cough in the acute phase is associated with subsequent physical and muscle weaknesses in the subacute phase.

## Introduction

Many countries have reported long-term health effects of coronavirus disease 2019 (COVID-19) ^(1), (2), (3)^. For example, a study in Japan reported persistent symptoms such as dyspnea, fatigue, cough, and dysosmia for >120 days after the COVID-19 onset ^(4)^. Old age, female sex, obesity, and presence of five or more symptoms in the acute phase of COVID-19 have been reported as risk factors associated with the persistent symptoms of COVID-19 ^(1)^. Furthermore, a Japanese investigation of COVID-19 sequelae in patients discharged after hospitalization between February 2020 and June 2020 identified sequelae in 48 of 63 enrolled patients (76%), with high rates of sequelae observed even among patients in their 20s (75%) and 30s (83%) ^(5)^. The definition of sequelae used in this previous investigation was determined according to symptomology that persisted for ≥14 days ^(5)^.

Nevertheless, the causes of persistent symptoms or sequelae in COVID-19 patients remain unclear, and treatment methods have yet to be established. Generally, patients affected by persistent symptoms or sequelae require assistance in returning to work, social life, and day-to-day life. In December 2021, Japan’s Ministry of Health, Labour, and Welfare issued guidelines termed “COVID-19 Medical Practice Guidelines, Supplementary Volume: Management of Post-COVID-19 Conditions (Provisional Version).” This document compiled information on best clinical practices concerning COVID-19 sequelae ^(6)^.

Fukui Prefecture has a population of approximately 760,000 and is located in central Japan, facing the Sea of Japan. The number of COVID-19 cases in this prefecture during the study period increased to 122 in the first period (March to June 2020), 122 in the second period (July to September 2020), 301 in the third period (October 2020 to February 2021), 876 in the fourth period (March to July 19, 2021), and 1,676 in the fifth period (March to July 19, 2021). Alpha- and delta-valiant strains were prevalent in the fourth and fifth trimesters ^(7)^. In Fukui Prefecture, ensuring that hospital beds are always available has facilitated a successful policy of early hospitalization for COVID-19 patients. Almost no patients have been forced to stay home and wait for a hospital bed after a COVID-19 diagnosis. This enabled early and timely treatment of COVID-19 and has thus more effectively prevented any adverse impacts due to delayed treatment. Analysis of the pathogenesis of COVID-19 in patients diagnosed in the current study will be useful for determining which medical activities to implement against COVID-19.

This study aimed to identify persistent and concomitant symptoms in patients admitted to the University of Fukui Hospital for COVID-19 based on a follow-up outpatient examination conducted 2 weeks after discharge. It also aimed to identify the same symptoms in the subacute phase in patients discharged from the hospital and to identify the risk factors for these symptoms. Furthermore, we present a brief literature review about the persistent symptomology of COVID-19, including studies of “long COVID” and “post-COVID-19.” This study was an exploratory investigation of the frequency of persistent symptoms in the subacute phase of COVID-19.

## Materials and Methods

### Study subjects and study duration

In this exploratory longitudinal study, we targeted consecutively presenting patients who were admitted to the University of Fukui Hospital for COVID-19 and discharged between April 3, 2020, and August 19, 2021. COVID-19 acceptance at the University of Fukui Hospital ranged from mild to moderate disease not requiring oxygen therapy. These patients then underwent a follow-up examination 2 weeks after discharge. During the study period, 151 COVID-19 patients were identified. Of these, 31 (30 patients who did not undergo the follow-up examination due to hospital transfer and one patient who did not meet the definition of patients without COVID-19 sequelae) were excluded from the current study, thus providing an analysis set of 120 patients (60 men and 60 women; mean age, 53.5 ± 17.0 years).

### Study methods and data collection

A technical assistant (a nurse) collected information on patient characteristics, date of COVID-19 onset, symptoms during the acute phase, and symptoms at the outpatient follow-up examination from medical records filed within the study period.

### Persistent and concomitant symptoms in the subacute phase of COVID-19 after hospital discharge

Persistent symptoms during the subacute phase of COVID-19 after hospital discharge were defined as symptoms that persisted for >14 days from the time of COVID-19 onset ^(5), (8)^. Furthermore, new concomitant symptoms that had appeared during the outpatient visit were included as persistent COVID-19 symptoms. At the time of the outpatient visit, any persistent, or concomitant symptom was considered a sequela of COVID-19.

### Ethics

This study was conducted with the approval of the ethics review committee and in accordance with the Declaration of Helsinki. All researchers involved in this study complied with the Ethical Guidelines for Medical and Biological Research Involving Human Subjects (MEXT/MHLW/METI Notification No. 1 of March 23, 2021).

### Opt-out

Because this study only evaluated existing information (i.e., patient medical records) and did not collect new samples or data, neither written, nor oral consent was obtained from the study subjects. This process was formally waived by the ethics review board at our medical center. Information about the study was disclosed to study subjects via the study webpage (http://research.hosp.u-fukui.ac.jp/rinsho/ethicscommittee/koukai_list/#chiiki_iryou). The study subjects were allowed to refuse participation in the study or to withdraw their consent for participation at any time.

### Statistical analysis

Statistical analysis was conducted using the EZR software (Saitama Medical Center, Jichi Medical University, Saitama, Japan; ver.1.54) ^(9)^. EZR is a statistical software that extends the functionality of R and R commander (The R Project for Statistical Computing, Vienna, Austria) ^(9)^. Continuous variables such as age, body mass index, and duration of follow-up are expressed as means ± standard deviations. Nominal (categorical) variables are expressed as the number of cases and frequencies (%) for each item. The two groups were compared using the Mann-Whitney U test for continuous variables and the χ2 test (including Yates continuity correction) for nominal variables. The three or four groups were compared using the Kruskal-Wallis test (multiple comparisons of two groups at a time with *post hoc* adjustment and Steel-Dwass multiple comparisons) for continuous variables and Fisher’s exact test (multiple comparisons of two groups at a time with Bonferroni adjustment) for nominal variables. Multivariate binomial logistic regression analysis with the presence or absence of COVID-19 sequelae as a dependent factor was employed to detect risk factors for COVID-19 sequelae. Clinical characteristics and acute-phase symptoms at admission were used as independent variables. To complement the analysis of acute-phase symptoms, we considered sputum expectoration related to cough and pharyngeal pain. The presence of both expectoration and cough symptoms was considered one factor. Both expectoration and pharyngeal pain were also analyzed as one factor. Independent variables were selected as factors exhibiting statistical or marginal significance in univariate analysis. Furthermore, independent variables that influenced each other were avoided in the selection. Multivariate analysis No. 1 was conducted using expectoration, nasal congestion, pharyngeal pain, dysosmia, dysgeusia, age (years), and sex (men) as independent variables. Multivariate analysis No. 2 was conducted using cough with expectoration, nasal congestion, pharyngeal pain, dysosmia, dysgeusia, age (years), and sex (men) as independent variables. Multivariate analysis No. 3 was conducted using pharyngeal pain with expectoration, nasal congestion, dysosmia, dysgeusia, age (years), and sex (men) as independent variables. In all analyses, a two-sided *p*-value of <0.05 was considered the threshold for statistical significance, and a *p*-value range of 0.05 ≤ p < 0.1 was considered as marginally significant.

## Results

### Clinical characteristics and changes in acute-phase symptoms at the time of admission

**Figure 1.**
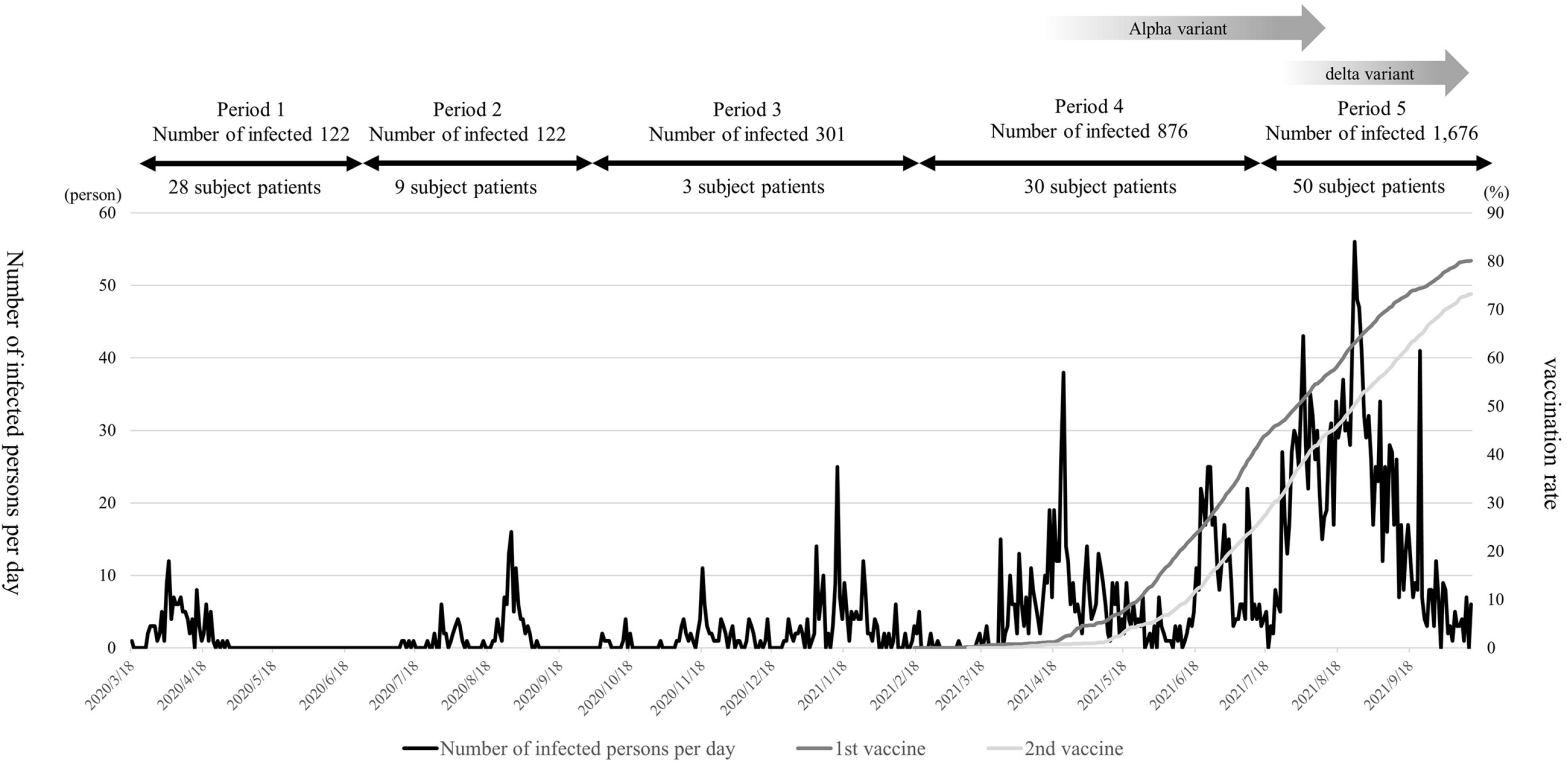
COVID-19 infection status and vaccination in Fukui Prefecture We present the status of COVID-19 infection in Fukui Prefecture from the first to the fifth period during this study. Changes in the first and second COVID-19 vaccination rates are also presented. The number of COVID-19 cases in Fukui Prefecture during the study period increased to 122, 122, 301, 876, and 1,676 in the first, second, third, fourth, and fifth periods, respectively. In Fukui Prefecture, the α variant was first identified on March 18, 2021, whereas the δ variant was first identified on July 17, 2021. The first round of COVID-19 vaccine in Fukui Prefecture began in February 2021 and the second in March 2021. By the end of the 5th period, the first vaccination coverage was 80.0%, and the second was 73.2%.

Clinical characteristics and changes in acute-phase symptoms at the time of admission are presented in Table 1. In addition, the trends of COVID-19 infection and vaccination status during the survey period in Fukui Prefecture are presented in Figure 1 ^(7), (10), (11)^. The most common acute symptom was pyrexia (90%), followed by cough (80.8%). Divided by the period of infection, the numbers of patients in this study were 28, 9, 3, 30, and 50 in the first, second, third, fourth, and fifth periods, respectively. In comparing the periods, there were significant changes in age (*p* < 0.001), pneumonia (*p* = 0.012), dysosmia (*p* = 0.034), dysgeusia (*p* = 0.023), and anorexia (*p* = 0.017). Also, smoking (*p* = 0.079), pharyngeal pain (*p* = 0.079), pharyngeal pain with expectoration (*p* = 0.065), myalgia (*p* = 0.059), and muscle weakness (*p* = 0.095) varied with marginal significance. Overall, only one patient was vaccinated against COVID-19.

**Table 1.** Clinical Characteristics and Changes in Acute-Phase Symptoms at the Time of Admission of COVID-19 Patients.

Clinical characteristics	Total n = 120	1st period ^a^ n = 28	2nd period ^b^ n = 9	3rd period ^c^ n = 3	4th period ^d^ n = 30	5th period ^e^ n = 50	*p*-value
Age (years)	53.5 ± 17.0	57.2 ± 14.1	74.7 ± 10.4	39.6 ± 19.8	52.1 ± 19.2	49.2 ± 14.8	<0.001^f^
Sex (men)	60 (50.0)	14 (50.0)	5 (55.5)	1 (50.0)	14 (46.6)	26 (52.0)	0.976
Body mass index (BMI) (kg/m^2^)	23.4 ± 4.0	23.7 ± 3.9	21.6 ± 3.4	21.8 ± 0.8	24.2 ± 4.0	23.2 ± 4.1	0.522
BMI (unknown)	2 (1.6)	-	-	-	1(3.3)	1(2.0)	-
Lifestyle habits
Smoking	23 (19.6)	3 (10.7)	0 (0.0)	1 (33.3)	4 (13.3)	15 (30.0)	0.079
Drinking alcohol	49 (40.8)	9 (32.1)	3 (33.3)	0 (0.0)	13 (43.3)	24 (48.0)	0.435
Underlying disease
Hypertension	40 (33.3)	14 (50.0)	3 (33.3)	1 (33.3)	6 (20.0)	16 (32.0)	0.182
Diabetes	18 (15.0)	5 (17.9)	2 (22.2)	0 (0.0)	3 (10.0)	8 (16.0)	0.825
Dyslipidemia	20 (16.7)	6 (21.4)	1 (11.1)	0 (0.0)	4 (13.3)	9 (18.0)	0.816
Hyperuricemia	11 (9.2)	3 (10.7)	1 (11.1)	0 (0.0)	4 (13.3)	3 (6.0)	0.799
Heart disease	11 (9.2)	1 (3.6)	2 (22.2)	0 (0.0)	2 (6.7)	6 (12.0)	0.419
Malignancy	9 (7.5)	3 (10.7)	0 (0.0)	1 (33.3)	2 (6.7)	3 (6.0)	0.376
Acute-phase symptoms
Cough	97 (80.8)	22 (78.6)	7 (77.8)	2 (66.7)	24 (80.0)	42 (84.0)	0.818
Expectoration	57 (47.5)	11 (39.3)	5 (55.6)	2 (66.7)	12 (40.0)	27 (54.0)	0.574
Pharyngeal pain	61 (50.8)	8 (28.6)	6 (66.7)	2 (66.7)	16 (53.3)	29 (58.0)	0.079
Cough with expectoration	52 (43.3)	9 (32.1)	5 (55.6)	1 (33.3)	11 (36.7)	26 (52.0)	0.375
Pharyngeal pain with expectoration	35 (29.2)	3 (10.7)	4 (44.4)	1 (33.3)	8 (26.7)	19 (38.0)	0.065
Respiratory discomfort	23 (19.2)	3 (10.7)	3 (33.3)	0 (0.0)	7 (23.3)	10 (20.0)	0.490
Pneumonia	84 (70.0)	23 (82.1)	8 (88.9)	0 (0.0)	23 (76.7)	30 (60.0)	0.012^g^
Runny nose	26 (21.7)	6 (21.4)	1 (11.1)	2 (66.7)	4 (13.3)	13 (26.0)	0.790
Nasal congestion	21 (17.5)	2 (7.1)	1 (11.1)	2 (66.7)	5 (16.7)	11 (22.0)	0.111
Pyrexia	108 (90.0)	25 (89.3)	7 (77.8)	2 (66.7)	4 (13.3)	13 (26.0)	0.122
Headache	38 (31.7)	5 (17.9)	3 (33.3)	1 (33.3)	11 (36.7)	18 (36.0)	0.442
Dysosmia	32 (26.7)	12 (42.9)	1 (11.1)	1 (33.3)	3 (10.0)	15 (30.0)	0.034^h^
Dysgeusia	29 (21.7)	11 (39.3)	1 (11.1)	1 (33.3)	2 (6.7)	14 (28.0)	0.023^i^
Abnormal vision	3 (2.5)	1 (3.6)	0 (0.0)	0 (0.0)	1 (3.3)	1 (2.0)	1.000
Arthralgia	15 (12.5)	4 (14.3)	0 (0.0)	0 (0.0)	3 (10.0)	8 (16.0)	0.794
Myalgia	9 (7.5)	0 (0.0)	0 (0.0)	0 (0.0)	6 (20.0)	3 (6.0)	0.059
Body pain	22 (18.3)	7 (25.0)	1 (11.1)	0 (0.0)	5 (16.7)	9 (18.0)	0.879
Muscle weakness	3 (2.5)	0 (0.0)	1 (11.1)	0 (0.0)	2 (6.7)	0 (0.0)	0.095
Physical weakness	1 (0.8)	0 (0.0)	0 (0.0)	0 (0.0)	0 (0.0)	1 (2.0)	1.000
Thirst	7 (5.8)	4 (14.3)	0 (0.0)	0 (0.0)	1 (3.3)	2 (4.0)	0.424
Diarrhea	47 (39.2)	10 (35.7)	2 (22.2)	1 (33.3)	8 (26.7)	26 (52.0)	0.147
Nausea	4 (3.3)	0 (0.0)	0 (0.0)	0 (0.0)	0 (0.0)	4 (8.0)	0.348
Anorexia	53 (44.2)	6 (21.4)	4 (44.4)	0 (0.0)	15 (50.0)	28 (56.0)	0.017^j^
Malaise	70 (58.3)	15 (53.6)	6 (66.7)	1 (33.3)	17 (56.7)	31 (62.0)	0.821

n (%), Mean ± standard deviation (unit) Fisher’s exact test was used for pairwise comparisons of categorical variables. The Kruskal-Wallis test was used for pairwise comparisons of continuous variables. The Bonferroni adjustment was used for multiple comparisons. f:a vs b *p* = 0.015, b vs d *p* = 0.031,b vs e *p* < 0.001; g:Fisher’s exact test revealed a significant difference overall, but multiple comparisons for each group using Bonferroni did not show a significant difference.; h:a vs d *p* = 0.064; i:a vs d *p* = 0.041;j:a vs e *p* = 0.042

### COVID-19 persistent and concomitant symptoms and changes in the clinical characteristics of patients after discharge from the hospital

COVID-19 changes in symptoms and clinical characteristics of patients after discharge are presented in Table 2. The most common acute symptom was physical weakness (48.4%), followed by muscle weakness (29.0%). Divided by the period of infection, the numbers of patients in this study were 13, 5, 10, and 34 in the first, second, fourth, and fifth periods, respectively. In comparing the periods, significant changes were observed in smoking (*p* = 0.034).

**Table 2.** Clinical Characteristics in the Post-Discharge and Changes in the Timing of Concomitant Symptoms of COVID-19 Patients.

Clinical characteristics	Total n = 62	1st period ^a^ n = 13	2nd period ^b^ n = 5	4th period ^c^ n = 10	5th period ^d^ n = 34	*p*-value
Age (years)	53.7 ± 15.9	55.8 ± 15.5	71.0 ± 11.7	55.7 ± 20.1	49.7 ± 13.9	0.060
Sex (men)	30 (48.3)	7 (53.8)	3 (60.0)	4 (40.0)	16 (47.0)	0.880
Body mass index (BMI) (kg/m^2^)	23.2 ± 4.3	24.0 ± 5.0	20.8 ± 3.4	23.9 ± 4.3	23.1 ± 4.2	0.566
BMI (unknown)	2 (3.2)	-	-	1 (10)	1 (2.9)	-
Lifestyle habits
Smoking	12 (19.3)	0 (0.0)	0 (0.0)	1 (10)	11 (32.4)	0.034^e^
Drinking alcohol	26 (41.9)	5 (38.5)	2 (40.0)	3 (30.0)	16 (47.1)	0.875
Underlying disease
Hypertension	19 (30.6)	6 (46.2)	1 (20.0)	2 (20.0)	10 (29.4)	0.627
Diabetes	11 (17.7)	2 (15.4)	2 (40.0)	1 (10.0)	6 (17.6)	0.584
Dyslipidemia	10 (16.1)	1 (7.7)	1 (20.0)	1 (10.0)	7 (20.6)	0.680
Hyperuricemia	6 (9.6)	1 (7.7)	1 (20.0)	1 (10.0)	3 (8.8)	0.826
Heart disease	8 (12.9)	0 (0.0)	2 (40.0)	1 (10.0)	5 (14.7)	0.139
Malignancy	6 (9.6)	2 (15.4)	0 (0.0)	2 (20.0)	2 (5.9)	0.349
concomitant symptoms

Physical weakness	30 (48.4)	6 (46.2)	4 (80.0)	3 (30.0)	17 (50.0)	0.348
Muscle weakness	18 (29.0)	4 (30.8)	3 (60.0)	3 (30.0)	8 (23.5)	0.384
Cough	10 (16.1)	1 (7.7)	0 (0.0)	4 (40.0)	5 (14.7)	0.171
Dysosmia	9 (14.5)	1 (7.7)	0 (0.0)	0 (0.0)	8 (23.5)	0.247
Dysgeusia	9 (14.5)	1 (7.7)	1 (20.0)	1 (10.0)	6 (17.6)	0.847
Respiratory discomfort	8 (12.9)	0 (0.0)	0 (0.0)	3 (30.0)	5 (14.7)	0.159
Malaise	5 (8.1)	0 (0.0)	0 (0.0)	0 (0.0)	5 (14.7)	0.362
Sleep disturbance	3 (4.8)	1 (7.7)	0 (0.0)	0 (0.0)	2 (5.9)	1.000
Pyrexia	2 (3.2)	1 (7.7)	0 (0.0)	0 (0.0)	1 (2.9)	0.703
Palpitation	2 (3.2)	1 (7.7)	0 (0.0)	0 (0.0)	1 (2.9)	0.703
Nasal congestion	1 (1.6)	1 (7.7)	0 (0.0)	0 (0.0)	0 (0.0)	0.452
Headache	1 (1.6)	1 (7.7)	0 (0.0)	0 (0.0)	0 (0.0)	0.452
Pharyngeal pain	1 (1.6)	1 (7.7)	0 (0.0)	0 (0.0)	0 (0.0)	0.452
Chilblain	1 (1.6)	1 (7.7)	0 (0.0)	0 (0.0)	0 (0.0)	0.452

n (%), Mean ± standard deviation (unit) Fisher’s exact test was used for pairwise comparisons of categorical variables. The Kruskal-Wallis test was used for pairwise comparisons of continuous variables. The Bonferroni adjustment was used for multiple comparisons. e:a vs b *p* = 0.015, b vs d *p* = 0.031,b vs e *p* < 0.001

### Comparison of COVID-19 with and without persistent and concurrent symptoms after discharge from the hospital

A comparison of the presence of persistent and concurrent symptoms of COVID-19 after discharge is presented in Table 3. Persistent symptoms of COVID-19 were observed in 62 patients (mean age, 53.7 ± 16.0 years; 48.4% men), representing 51.7% of all patients enrolled in this investigation. The time from the onset of COVID-19 to the confirmation of COVID-19 sequelae in the subjects was 34.2 ± 8.7 days. Patients with persistent COVID-19 symptoms after discharge from the hospital had a significantly higher incidence of expectoration, cough with expectoration, and dysgeusia on admission (acute phase) compared with patients without persistent symptoms (expectoration: 59.3% vs 36.1%, *p* = 0.0179; cough with expectoration: 66.0% vs 29.3%, *p* = 0.00146; dysgeusia: 32.3% vs 15.5%, *p* = 0.0254). Marginal significance also included differences in pharyngeal pain with expectoration, nasal congestion, myalgia, and dysosmia (pharyngeal pain: 59.7% vs 41.4%, *p* = 0.068; pain with expectoration: 40.4% vs 22.0%, *p* = 0.075; nasal congestion: 24.2% vs 10.3%, *p* = 0.079; dysosmia: 37.1% vs 15.5%, *p* = 0.053).

**Table 3.** Comparison of COVID-19 Patients with and without Concurrent Symptoms after Discharge from Hospital.

	Overall n = 120	Patients with COVID-19 sequelae n = 62	Patients without COVID-19 sequelae n = 58	*p*-value
Age, years n (%)	53.5 ± 17.0	53.7 ± 16.0	53.3 ± 18.2	0.858
Sex, men n (%)	60 (50.0)	30 (48.4)	30 (51.7)	0.855
Body mass index (BMI) (kg/m^2^)	23.4 ± 4.0	23.3 ± 4.4	23.6 ± 3.6	0.183
BMI, unknown n (%)	2 (1.6)	-	-	-
Lifestyle habits
Smoking n (%)	23 (19.6)	12 (19.4)	11 (19.0)	1
Drinking alcohol n (%)	49 (40.8)	26 (41.9)	23 (39.7)	0.946
Underlying disease
Hypertension n (%)	40 (33.3)	19 (30.6)	21 (36.2)	0.651
Diabetes n (%)	18 (15.0)	11 (17.7)	7 (12.1)	0.539
Dyslipidemia n (%)	20 (16.7)	10 (16.1)	10 (17.2)	1
Hyperuricemia n (%)	11 (9.2)	6 (9.7)	5 (8.6)	1
Heart disease n (%)	11 (9.2)	8 (12.9)	3 (5.2)	0.25
Malignancy n (%)	9 (7.5)	5 (8.5)	4 (6.6)	0.959
Acute-phase symptoms

Cough n (%)	97 (80.8)	54 (87.1)	43 (74.1)	0.116
Expectoration n (%)	57 (47.5)	35 (59.3)	22 (36.1)	0.0179
Pharyngeal pain n (%)	61 (50.8)	37 (59.7)	24 (41.4)	0.0686
Cough with expectoration n (%)	52 (43.3)	36 (66.0)	16 (29.3)	0.00146
Pharyngeal pain with expectoration n (%)	35 (29.2)	23 (40.4)	12 (22.0)	0.0759
Pneumonia n (%)	84 (70.0)	44 (71.0)	40 (69.0)	0.968
Runny nose n (%)	26 (21.7)	16 (25.8)	10 (17.2)	0.359
Nasal congestion n (%)	21 (17.5)	15 (24.2)	6 (10.3)	0.0793
Pyrexia n (%)	108 (90.0)	59 (95.2)	49 (84.5)	0.1
Headache n (%)	38 (31.7)	24 (38.7)	14 (24.1)	0.129
Dysosmia n (%)	32 (26.7)	23 (37.1)	9 (15.5)	0.0539
Dysgeusia n (%)	29 (21.7)	20 (32.3)	9 (15.5)	0.0254
Abnormal vision n (%)	3 (2.5)	2 (3.2)	1 (1.7)	0.1
Arthralgia n (%)	15 (12.5)	9 (14.5)	6 (10.3)	0.679
Myalgia n (%)	9 (7.5)	5 (8.1)	4 (6.9)	1
Body pain n (%)	22 (18.3)	14 (22.6)	8 (13.8)	0.314
Muscle weakness n (%)	3 (2.5)	1 (1.6)	2 (3.4)	0.953
Physical weakness n (%)	1 (0.8)	1 (1.6)	0 (0)	1
Thirst n (%)	7 (5.8)	5 (8.5)	2 (3.3)	0.491
Diarrhea n (%)	47 (39.2)	28 (45.2)	19 (32.8)	0.229
Nausea n (%)	4 (3.3)	3 (4.8)	1 (1.7)	0.659
Anorexia n (%)	53 (44.2)	29 (46.8)	24 (41.4)	0.681
Malaise n (%)	70 (58.3)	41 (66.1)	29 (50.8)	0.108

n (%), mean ± standard deviation (unit) Continuous variables: Mann-Whitney U test, Nominal variables: 2 test (including Yates continuity correction)

### Risk factors associated with COVID-19 sequela (persistent and concomitant symptoms)

Risk Factors Associated with COVID-19 Sequela is presented in Table 4.

**Table 4.** Risk Factors Associated with COVID-19 Persistent and Concomitant Symptoms.

	Multivariate analysis No.1			Multivariate analysis No.2				Multivariate analysis No.3			
	OR	95% CI lower-upper	*p*-value		OR	95% CI lower-upper	*p*-value		OR	95% CI lower-upper	*p*-value
Expectoration	2.36	1.070-5.190	0.033	Cough with expectoration	2.94	1.300-6.630	0.009	Pharyngeal pain with expectoration	2.20	0.926-5.200	0.074
Nasal congestion	2.00	0.611-6.510	0.252	Nasal congestion	2.00	0.607-6.590	0.254	Nasal congestion	2.32	0.720-7.450	0.159
Pharyngeal pain	1.79	0.809-3.980	0.151	Pharyngeal pain	1.68	0.750-3.780	0.207	Dysosmia	2.36	0.760-7.310	0.137
Dysosmia	2.47	0.778-7.820	0.125	Dysosmia	2.23	0.698-7.140	0.176	Dysgeusia	1.75	0.566-5.380	0.332
Dysgeusia	1.60	0.509-5.030	0.422	Dysgeusia	1.66	0.520-5.300	0.392	Age (years)	1.02	0.991-1.040	0.212
Age (years)	1.01	0.989-1.040	0.255	Age (years)	1.01	0.987-1.040	0.333	Sex (men)	1.18	0.573-2.600	0.6770
Sex (men)	1.10	0.493-2.480	0.807	Sex (men)	1.05	0.464-2.380	0.905	-	-	-	-

Multiple logistic regression analysis (binomial logistic regression analysis); CI: confidence interval

#### Multivariate analysis No. 1

Binomial logistic regression analysis revealed expectoration in the acute phase (odds ratio [OR] 2.36, 95% confidence interval [CI]: 1.070-5.190, *p* = 0.033) as a risk factor predisposing patients to COVID-19 sequelae.

#### Multivariate analysis No. 2

Binomial logistic regression analysis revealed cough with expectoration in the acute phase (odds ratio [OR] 2.96, 95% CI: 1.300-6.630, *p* = 0.0094) as a risk factor predisposing patients to COVID-19 sequelae.

#### Multivariate analysis No. 3

Binomial logistic regression analysis failed to indicate a factor as an independent variable.

### Literature review: Frequencies and risk factors for persistent symptoms

We found a total of four articles that reported on the frequency of persistent symptoms and associated risk factors ^(1), (12), (13), (14)^ and nine articles that reported on the frequency of persistent symptoms only ^(2), (4), (5), (9), (15), (16), (17), (18), (19), (20)^. Representative data from these 12 studies as well as the present study are presented in Table 5.

**Table 5.** Frequency of Persistent Symptoms, Risk Factors, and Past Reports in COVID-19 Patients.

This study and references	Country where the research was conducted	Study period	Target group	Number of subjects	Sex (Men)	Age (years)	Duration studied (after onset)	Frequency	Most common symptom	Second most common symptom	Risk factor(s)
Study	Japan	April 2020-August 2021	Hospitalized	120	50%	53.5 ± 17.0	Mean, 34.2 days	51.70%	Physical weakness: 48.4%	Muscle weakness: 29.0%	Expectoration with cough (in the acute phase)
Tokyo iCDC Expert Board Infectious Disease Care Team ^(5)^	Japan	Februar-June 2020	Hospitalized	63	66.7%	48.1 ± 18.5	14 days	76%	non	non	non
Miyazato et al. ^(4)^	Japan	February-June 2020	Hospitalized	63	66.7%	48.1 ± 18.5	After 60 days	non	Dysosmia: 19%	Reparatory discomfort: 18%	non
							After 120 days	non	Respiratory discomfort: 11%	Dysosmia: 10% Lassitude: 10%	
Petersen et al. ^(12)^	Faroe Islands	April-June 2020	Nonhospitalized	180	46.0%	39.9 (0-3)	Mean, 125 days	53.0%	Malaise	Weakened sense of smell	non
Huang et al. ^(1)^	China	Jan-May 2020	Hospitalized	1,733	52%	57 (47-5)	After 6 months	76%	Fatigue with muscle weakness: 63%	Sleep disturbance: 26%	Old age, female sex, obesity, 5 or more symptoms in the acute phase
Taquet et al. ^(16)^	United States of America	July-December 2020	COVID-19 infected	273,618	44.4%	46.3 ± 19.8	1-180 days	57% (including acute-phase symptoms)	Anxiety/depression: 22.8%	Abnormal respiration: 18.7%	Severe COVID-19, female sex, young adult
							90-80 days	36.5%	Anxiety/depression: 15.5%	Abdominal symptom: 8.3%	
Logue et al. ^(13)^	United States of America	August-November 2020	Hospitalized (9.0%) Nonhospitalized (84.7%)	177	42.9%	48.0(18-4)	Mean, > 6 months	31.1%	Malaise: 13.6%	Dysosmia/dysgeusia: 13.6%	non
Peghin et al. ^(18)^	Italy	March-May 2020	Hospitalized (26.2%) Nonhospitalized (73.7%)	599	46.6%	53 ± 15.8	After 6 months	40.2% (outpatients: 35.5%, inpatients: 53.5%)	Malaise	Dysosmia/dysgeusia	Female sex, number of symptoms (proportional increase), admitted to the ICU
Nehme et al. ^(14)^	Switzerland	March-May 2020	Nonhospitalized	410	32.9%	42.7 ± 12.9	After 7-9 months	39.0%	Malaise: 20.7%	Dysosmia/dysgeusia: 16.8%	non
Liu et al. ^(2)^	China	February-April 2020	Hospitalized	502 (traceable)	46.3% (594 subjects)	63 (53-68) (594 subjects)	After 3 months	51.2%	Insomnia: 16.9%	Tightness of the chest: 15.3%	non
				422 (Traceable)			After 6 months	40.0%	Chest tightness: 12.6%	Malaise: 6.4%	
Huang et al. ^(17)^	China	Jan- May 2020	Hospitalized	1,276	53%	59(49-7)	After 6 months	68%	Malaise or muscle weakness: 52.0%	Sleep disturbance:27.0%	non
							After 12 months	49%	Malaise or muscle weakness: 20.0%	Sleep disturbance:17.0%	
Asadi-Pooya et al. ^(20)^	Iran	February 2020-March 2021	Hospitalized	2,685	non	non	3-6 months	66%	Fatigue: 32%	Exercise intolerance: 26%	non
				1,996			6-12 months	57.1%	Fatigue: 25%	Exercise intolerance: 20%	

## Discussion

This study focused on standard literature search databases such as PubMed, which were used to find studies on the frequency of persistent symptoms of COVID-19 (such symptomology is sometimes termed “long COVID” or “post-COVID syndrome”), examined the frequency of these persistent symptoms as well as their associated risk factors, and compared our findings with the results of existing reports.

More than half of the patients in this study had persistent and concomitant symptoms. The most common of these persistent and concomitant symptoms was a decline in physical function, with physical, and muscle weaknesses being the most common findings. Based on the study duration, it is reasonable to conclude that these persistent symptoms commonly occur in the subacute phase with COVID-19 sequela (persistent and concomitant symptoms).

COVID-19 is often compared with influenza, a respiratory infection lasting 2-8 days and causing a range of symptoms such as cough, pyrexia, myalgia, chills, sweating, and malaise ^(21)^. In this study, symptoms lasting 34.2 ± 8.7 days were observed in at least half of the enrolled patients, indicating that COVID-19 symptoms may persist for a longer period than the influenza symptoms.

In existing reports on persistent COVID-19 symptoms, the proportion of patients with persistent symptoms at 14 days from the time of disease onset (the shortest duration studied) was 76%, and the proportion of patients with persistent symptoms at 12 months from disease onset (the longest duration studied) was 49% ^(5), (17)^. Differences in study duration must be considered when comparing the frequency of persistent symptoms across studies, as must differences in subject characteristics. For example, studies that collected data after 6 months found that the proportion of patients with persistent symptoms widely ranged from 40.0% to 76.0% ^(1), (2), (17), (18)^. This variability may be caused by the different severities of COVID-19 and the different times of hospital admission among the studied subjects and may also be influenced by how each study defines “persistent symptoms” of COVID-19.

The World Health Organization (WHO) published a definition of “post-COVID-19 condition” on October 6, 2021, as follows ^(22)^: “post-COVID-19 condition occurs in individuals with a history of probable or confirmed SARS-CoV-2 infection, usually three months from the onset of COVID-19 with symptoms that last for at least two months and cannot be explained by an alternative diagnosis ^(22)^.” One of the reasons why studies published to date have examined different periods and have applied different definitions of persistent symptoms is that those investigations were conducted before the WHO published their definition.

Huang et al. found that the more severe the illness during the acute phase, the greater the proportion of patients with impaired respiratory function at a later date ^(1)^. The severity of COVID-19 in the acute phase among the studied subjects must also be considered. A major difference between this study and the previous ones is that delayed treatment had no impact on the persistent symptoms of COVID-19 in our study. In Fukui Prefecture, all people who have confirmed positive findings for SARS-CoV-2 must be hospitalized as a precaution against the spread of infection. Hence, all patients enrolled in this study were hospitalized early in their disease course. Furthermore, because cooperation between hospitals, and the local government in this prefecture ensures that hospital beds are always available, all patients receive early essential treatment for COVID-19. Nevertheless, even with early intervention, persistent symptoms of COVID-19 were observed in a high proportion of patients (51.7%).

Two multivariate analyses were conducted in this study. Expectoration (OR: 2.36) was a risk factor in the first analysis, and cough with expectoration (OR: 2.96) was a risk factor in the second analysis. Because the OR was higher in cases with expectoration and cough than with expectoration alone, we assume that cases with expectoration and cough are more important. In this study, we found that cough with expectoration in the acute phase of COVID-19 is a risk factor for persistent and concomitant symptoms occurring in the subacute phase after hospital discharge. SARS-CoV-2 proliferates in both the upper and lower respiratory tract, with particularly high viral loads observed in the upper respiratory tract ^(23)^. The main symptoms of COVID-19 are pyrexia and respiratory symptoms (e.g., cough) ^(24)^. Coughing is a protective reflex intended to remove foreign substances and airway secretions from the airways and is an important biological response ^(25)^. A previous study that investigated energy expenditure due to coughing showed an increase in energy expenditure of 11% from 2.72 ± 0.60 mL/kg/min at rest to 3.03 ± 0.62 mL/kg/min while coughing ^(26)^. Increased coughing due to respiratory infection also increases energy expenditure, which is very draining on the body. Furthermore, when coughing continues through the night, it can lead to sleep deprivation and delayed recovery. More than 70% of persistent symptoms observed in this study were manifestations of weakened physical function (physical and muscle weaknesses), indicating a possible relationship between persistent symptoms and physical exhaustion due to coughing and expectoration during the acute phase of COVID-19. In addition, although this study did not assess psychological effects, the long-term psychological impact of stress arising from disease-associated anxiety as well as the restrictions on movement associated with COVID-19 must be considered in interpreting our findings.

To the best of our knowledge, no established methods are available for the treatment of COVID-19 sequelae, and treatment instead mainly focuses on symptom management ^(6)^. The UK Health Security Agency has reported that people who have received one or more doses of a COVID-19 vaccine are less likely to develop Long-term effects of COVID-19 (long COVID) or post-COVID-19 syndrome than those who are unvaccinated ^(27)^. Only one patient in this study was vaccinated for COVID-19. If vaccinated patients had been included in this study, the frequency of persistent symptoms may have been lower. Furthermore, if COVID-19 vaccines are effective at preventing COVID-19 sequelae within rigorous, highly powered research, this would add to the evidence base supporting the importance of vaccines.

COVID-19 symptoms are thought to vary with the emergence of new variants and the status of immunity from vaccines and other sources ^(28)^. Also, the sequelae, and long-term manifestations of COVID-19 differ in symptoms and frequency of manifestations by the SARS-CoV-2 variants ^(29)^. The COVID-19 strains in this study include the conventional strain, α variant, and δ variant. In this study, the differences in symptoms during the acute phase were more pronounced in pneumonia, olfactory and taste disorders, and anorexia. However, the results are considered as reference information due to the small number of subjects in the second and third phases. There were no differences in persistent or concomitant symptoms at each time point. The number of patients in each study period does not allow for a clear comparison of symptoms.

### Limitations

This study had some limitations. First, for the explanation of the association between cough with expectoration in the acute phase and COVID-19 sequelae, we cannot estimate the exact causality. However, cough with expectoration in the acute-phase disability may be the reason for future COVID-19 sequelae after discharge. Second, this was a single-center study with a small sample size. Third, these findings may not be limited to COVID-19. Fourth, this study may have suppressed certain information due to interviewer bias. Fifth, physical information is not available in the form of body or muscle strength measurements. Sixth, the COVID-19 mutant strain of the subjects was unknown. The sixth aforementioned limitations should be considered in future studies.

In conclusion, we observed persistent, and concomitant symptoms of COVID-19 in the subacute phase in 51.7% of patients at hospital discharge. These symptoms include physical and muscle weaknesses, suggesting that persistent, and concomitant symptoms of COVID-19 were caused by physical fatigue induced by coughing. When comparing our findings with those of previous studies, differences in how persistent COVID-19 symptoms are defined must be considered. Our findings inform future research directions and directly inform medical guidelines and clinical decision-making.

## Article Information

### Conflicts of Interest

None

### Acknowledgement

We thank Editage (www.editage.com) for writing support.

### Author Contributions

All authors meet the ICMJE authorship criteria and contributed to the intellectual content of the manuscript. TK and HO designed the study and wrote the first draft of the manuscript. TK, HO, and OY were responsible for the data analysis. HH was responsible for the organization and coordination of the study. OY, IS, HI, and HH assisted in revising the manuscript. All authors are responsible for the interpretation of data and agree to be accountable for all aspects of the work in ensuring that questions related to the accuracy or integrity of any part of the work are appropriately investigated and resolved. All authors critically revised the manuscript for important intellectual content and approved the final version.

### Data Sharing

All data underlying the findings are within the paper.

### Approval by Institutional Review Board (IRB)

This study was conducted with the approval of the University of Fukui Medical Research Ethics Review Committee (Approval No.: 20210098). This study was conducted in accordance with the Declaration of Helsinki. All researchers involved in this study complied with Ethical Guidelines for Medical and Biological Research Involving Human Subjects (MEXT/MHLW/METI Notification No. 1 of March 23, 2021). Because this study only evaluated existing information (i.e., patient medical records) and did not collect new samples or data, neither written nor oral consent was obtained from the study subjects. The study subjects were allowed to refuse participation in the study or to withdraw their consent for participation at any time. This process was formally waived by the ethics review board at our medical center. Information about the study was disclosed to study subjects via the study webpage (http://research.hosp.u-fukui.ac.jp/rinsho/ethicscommittee/koukai_list/#chiiki_iryou).
